# Nonstructural Protein NS1 of Influenza Virus Disrupts Mitochondrial Dynamics and Enhances Mitophagy via ULK1 and BNIP3

**DOI:** 10.3390/v13091845

**Published:** 2021-09-15

**Authors:** Jae-Hwan Lee, Soo-Jin Oh, Jeanho Yun, Ok Sarah Shin

**Affiliations:** 1 BK21 Graduate Program, Department of Biomedical Sciences, College of Medicine, Korea University Guro Hospital, Seoul 08308, Korea; dlwo4949@gmail.com (J.-H.L.); sjooooh@gmail.com (S.-J.O.); 2 Peripheral Neuropathy Research Center, Department of Translational Biomedical Sciences, College of Medicine, Dong-A University, Busan 49201, Korea

**Keywords:** influenza a virus, NS1, mitophagy, antiviral immune responses, BNIP3

## Abstract

Nonstructural protein 1 (NS1) of influenza virus (IFV) is essential for evading interferon (IFN)-mediated antiviral responses, thereby contributing to the pathogenesis of influenza. Mitophagy is a type of autophagy that selectively removes damaged mitochondria. The role of NS1 in IFV-mediated mitophagy is currently unknown. Herein, we showed that overexpression of NS1 protein led to enhancement of mitophagy. Mitophagy induction via carbonyl cyanide 3-chlorophenylhydrazone treatment in IFV-infected A549 cells led to increased viral replication efficiency, whereas the knockdown of PTEN-induced kinase 1 (PINK1) led to the opposite effect on viral replication. Overexpression of NS1 protein led to changes in mitochondrial dynamics, including depolarization of mitochondrial membrane potential. In contrast, infection with NS1-deficient virus resulted in impaired mitochondrial fragmentation, subsequent mitolysosomal formation, and mitophagy induction, suggesting an important role of NS1 in mitophagy. Meanwhile, NS1 protein increased the phosphorylation of Unc-51-like autophagy activating kinase 1 (ULK1) and the mitochondrial expression of BCL2- interacting protein 3 (BNIP3), both of which were found to be important for IFV-mediated mitophagy. Overall, these data highlight the importance of IFV NS1, ULK1, and BNIP3 during mitophagy activation.

## 1. Introduction

Mitophagy, a selective autophagy of mitochondria, promotes mitochondrial quality control by inducing the clearance of damaged mitochondria via the autophagic machinery [1,2]. Defective mitophagy is associated with impaired energy metabolism and unwanted chronic systemic inflammation, thus contributing to the pathogenesis of inflammatory and autoimmune diseases [3]. During mitophagy, impaired mitochondria can be tagged by the kinase PTEN-induced putative kinase protein 1 (PINK1), which triggers the recruitment of the E3-ubiquitin ligase Parkin, followed by the subsequent mitochondrial sequestration within autophagosomes. Accumulating evidence suggests that mitophagy is modulated by multiple viruses to subvert and evade the innate immune response [4]. For example, influenza viruses can directly trigger mitophagy via their own viral components [5,6].

Influenza is a major infectious disease leading to annual seasonal epidemics and sporadic pandemics, which cause high morbidity and mortality in humans [7]. Influenza viruses (IFV) belong to the *Orthomyxoviridae* family and contain eight RNA segments, which encode RNA polymerase units, hemagglutinin (HA), neuraminidase (NA), viral nucleoprotein (NP), matrix protein (M1), membrane protein (M2), non-structural protein NS1, and nuclear export protein. Given the segmented nature of the IFV genome, IFV can undergo genome reassortment and circulate between species. Oseltamivir is one of currently available anti-influenza drugs, which target the NA active sites, and have been widely used for more than 10 years; however, during the 2007–2008 influenza season, cases of oseltamivir-resistant IFV A H1N1 emerged rapidly [8], highlighting the need to determine novel targets that can be exploited to inhibit viral replication and prevent the spread of IFV infections.

IFV NS1 is a multifunctional protein that plays a major role in antagonizing antiviral IFN responses [9,10]. RIG-I-like receptors, including retinoic acid-inducible gene-I (RIG-I) and melanoma differentiation-associated gene 5, typically recognize RNA viruses and lead to interaction with mitochondrial antiviral signaling protein (MAVS) to induce TBK1-dependent activation of interferon regulatory factor 3, which upon activation, is translocated to the nucleus for transcriptional activation of IFNs [11]. As an IFN antagonist, NS1 blocks the antiviral response at multiple stages of the IFN signaling cascade. In particular, NS1 mitigates IFN induction by inhibiting RIG-I-ubiquitination mediated by tripartite motif-containing (TRIM) 25 protein, which is essential for the robust induction of type I and III IFN production [12]. NS1 protein also interferes with interferon stimulated gene (ISG) activities, including protein kinase R and oligoadenylate synthase [13]. Additionally, NS1 targets phosphoinositide 3-kinase (PI3K) signaling pathways by direct interaction with the PI3K p85b subunit, thereby playing a key role in anti-apoptotic pathways [14]. In addition to its anti-apoptotic role, Zhirnov et al., suggested that IFV NS1 can stimulate autophagy indirectly by upregulating the synthesis of HA and M2 [15]. The role of autophagy during IFV infection has been extensively studied by multiple groups [16,17,18]. However, whether the IFV NS1 protein directly or indirectly mediates IFV-mediated mitophagy and affects innate immune evasion is currently unknown.

In this study, we investigated IFV-induced mitochondrial dynamics in A549 cells and the potent role of the NS1 protein during mitophagy. We observed that CCCP-mediated mitophagy facilitates IFV replication. Furthermore, NS1 plays an important role in triggering mitochondrial fission and subsequent mitophagy. Altogether, our data indicate that IFV NS1 may utilize mitophagy to evade innate immune responses.

## 2. Materials and Methods

### 2.1. Cells, Viruses, and Reagents

A549 cells were cultured at 37 °C in RPMI 1640 medium (Corning Mediatech, Corning, NY, USA) supplemented with 10% fetal bovine serum (FBS; Corning Mediatech) and 1% antibiotics. HEK293T and HeLa cells were cultured at 37 °C in Dulbecco’s modified Eagle’s medium (DMEM; Corning Mediatech) supplemented with 10% FBS and 1% antibiotics. HeLa cells stably expressing the mitochondria-targeted fluorescent protein Keima (mt-Keima) [19,20,21] were used to quantitatively measure mitophagy activity via confocal microscopy.

Human IFV A/Puerto-Rico/8/34 (H1N1) PR8 and recombinant IFV with NS1 deletion (PR8dNS1) were generated as described previously (a kind gift from Dr. Adolfo Garcia-Sastre, Icahn School of Medicine at Mount Sinai, NY, USA) [22]. Virus titers in MDCK cells were determined using a standard plaque assay, with minor modifications [23]. For viral infection, cells were infected with IFV at the multiplicity of infection (MOI) of 1 in a minimal volume of media supplemented with 0.5 mg/mL TPCK-treated trypsin (Sigma-Aldrich, St. Louis, MO, USA) and 2% FBS. After 90 min, cells were incubated with fresh infection medium at 37 °C for the indicated times. Virus titers in supernatants were measured by the TCID50 assay performed on MDCK cells.

Carbonyl cyanide m-chlorophenyl hydrazone (CCCP), chloroquine (CQ), rapamycin, 3-MA, SBI-0206965 (ULK1 inhibitor), and dimethyl sulfoxide (DMSO) were purchased from Sigma-Aldrich (St. Louis, MO, USA). The cells were treated with various drug doses prior to infection. LysoTracker Red and MitoTracker Green dyes were purchased from Thermo Fisher Scientific, Waltham, MA, USA.

### 2.2. Confocal Microscopy and Image Analysis

The IFV NS1-FLAG plasmid was purchased from Sino Biological. LC3-GFP, Parkin-GFP, and mito-dsRED plasmids were previously described [21]. Cells were seeded onto coverslips in 24-well plates and transfected with various plasmids, followed by IFV infection at various time points. Cells were washed with PBS, fixed with 4% paraformaldehyde, and permeabilized with 0.1% Triton X-100, as described previously [24]. NS1-FLAG protein was detected by treatment with mouse anti-FLAG tag antibodies (Wako Pure Chemical Industries, Osaka, Japan), followed by treatment with a secondary goat anti-mouse IgG (H+L) cross-adsorbed secondary antibody, Cascade Blue (Thermo Fisher Scientific). Coverslips were mounted on glass slides using mounting media containing 4,6-diamidino-2-phenylindole (DAPI). The images were examined using Zeiss LSM900 confocal microscope and analyzed by ZEN software as previously described [25,26].

### 2.3. Quantitative Realtime PCR (qRT-PCR)

Total RNA was extracted using TRIzol reagent (Invitrogen, Carlsbad, CA, USA) as described previously [27,28] and then was reverse transcribed into cDNA using ImProm-II Reverse Transcription System (Promega, Madison, WI, USA). The mRNA expression of genes was quantified by real-time PCR using Power SYBR^®^ Green Master Mix (Thermo Fisher Scientific) on the QuantStudio 6 Flex Real-time PCR system. β-actin was used as an internal reference for normalizing the gene expression quantified by real-time PCR. The primer sequences are listed in the Table 1 [29].

### 2.4. Small Interfering RNA (siRNA) Transfection

Human control siRNA, PINK1-specific siRNA, BNIP3-specific siRNA, and BNIP3L-specific siRNA were obtained from Bioneer, Daejeon, Korea and sequences are provided in Table 2. For siRNA transfection, cells were plated into 6 well plates and incubated overnight. For 20 min at room temperature, 100 pmol of siRNAs were mixed with 1:2 ratio of RNAiMAX reagent (Invitrogen) according to the manufacturer’s protocol, then was added into a matching well. PINK1, BNIP3, and BNIP3L gene expression levels were quantified by qRT-PCR.

### 2.5. Mitochondrial Membrane Potential (MMP) Measurements

MMP was measured using the fluorescent dye tetramethylrhodamine methyl ester (TMRM; T668; Thermo Fisher Scientific). Cells cultured in 96-wells plates were washed once with PBS and incubated with 200 nM TMRM at 37 °C for 30 min. TMRM fluorescence was measured using a Varioskan LUX multimode microplate reader (Thermo Fisher Scientific) and the SkanIt Software 5.0, with excitation and emission wavelengths of 548 nm and 573 nm, respectively.

### 2.6. Mitochondrial Isolation and Immunoblot Analysis

The mitochondrial fractions were isolated using the Mitochondria Isolation Kit for Cultured Cells (Abcam) according to manufacturer’s instruction. Immunoblot analysis was performed as described previously [29,30]. Cells were lysed with RIPA buffer (Sigma-Aldrich) mixed with a protease and phosphatase inhibitor cocktail (Roche) at the specified time points. Samples were subjected to SDS-PAGE gels, transferred onto polyvinylidene difluoride membranes (Millipore), and blocked with 5% skim milk in Tris-buffered saline buffer supplemented with 0.1% Tween-20 (TBS-Tw) for 1 h at room temperature. This was followed by overnight incubation at 4 °C with the following primary antibodies: anti-phospho-AKT (S473) (4060), anti-cleaved caspase-3 (Asp175) (9664), anti-caspase 3 (9662), anti-cleaved PARP (Asp214) (5625), anti-PARP (9542), anti-phospho-ULK1 (Ser757) (6888), anti-ULK1 (8054), anti-OPTN (58,981), anti-BNIP3 (44,060), anti-BNIP3L (12,396), anti-p62 (8025), anti-LC3B (3868), anti-TOM20 (42,406), anti-Calnexin (2679) were purchased from Cell Signaling Technology, whereas Mouse anti-FLAG antibody (018-2381) was purchased from Wako and IFV NP1 and M1 antibodies were obtained from GeneTex and Sino biological, respectively. Anti-IFV NS1 antibody was purchased from Santa Cruz Biotechnology. Tubulin or β-actin (Abgent, CA, USA) antibody was used as the loading control. After three washes in TBS/Tw, the membranes were incubated with horseradish peroxidase-conjugated anti-rabbit or anti-mouse IgG secondary antibodies for 1 h at 25 °C. Membranes were then washed with TBS-Tw and incubated with ECL Western Blotting Substrate (Thermo Fisher Scientific). Representative images of three independent experiments are shown.

### 2.7. Apoptosis Assay

For detecting cell apoptosis, A549 cells were collected and incubated with the FITC-conjugated Annexin V apoptosis detection kit (BD Bioscience, Franklin Lakes, NJ, USA) according to the manufacturer’s protocol and the stained cells were analyzed using a BD LSR Fortessa™ X-20 Cell Analyzer (BD Bioscience, Piscataway, NJ, USA) [29].

### 2.8. Statistical Analysis

The results are expressed as the mean ± standard deviation (SD). The data were analyzed using Mann–Whitney test or Student’s *t*-test to determine the significance of the difference between the two groups. Statistical analyses were performed using Prism 7.0 software (Graphpad Inc., San Diego, CA, USA).

## 3. Results

### 3.1. IFV NS1 Promotes CCCP-Mediated Mitophagy in A549 Cells

IFV NS1 protein is known to participate in a wide range of functions, modulating cell cycles and antagonizing antiviral host cellular responses [10]. To confirm this, we overexpressed IFV NS1 in A549 cells and determined the effect of NS1 on apoptosis. Staurosporine, an apoptosis inducer that activates caspase-3, was added to the cells, and the protein expression levels of cleaved caspases and PARP were detected by immunoblot analysis. NS1-overexpressing cells showed diminished expression levels of cleaved caspase 3 and PARP compared with empty vector (EV)-overexpressing cells, suggesting NS1-driven protection against apoptosis (Supplementary Figure S1A). Meanwhile, NS1 overexpression led to increased AKT phosphorylation, as reported previously [31]. The anti-apoptotic effect of the IFV NS1 protein was further confirmed by flow cytometry analysis using annexin-V5/propidium iodide staining. An increased number of cells undergoing apoptosis was found in NS1-deficient virus-infected cells, compared to those in PR8-infected cells (Supplementary Figure S1B).

**Figure 1:**
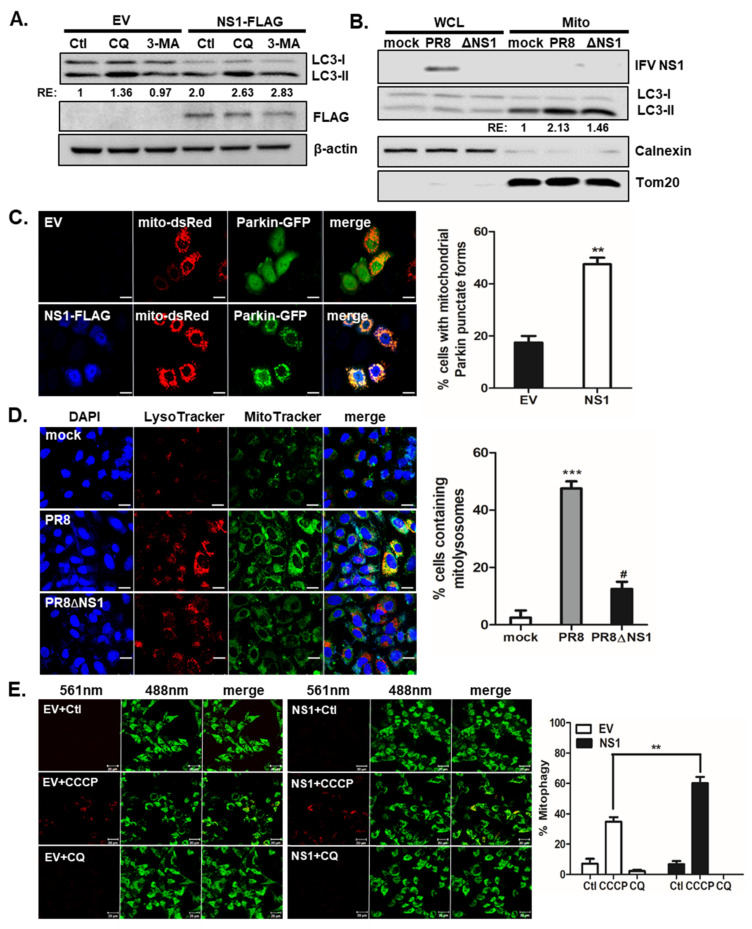
IFV NS1 promotes CCCP-mediated mitophagy. (**A**) Cells were empty vector (EV) or NS1-FLAG-transfected for 24 h and then treated with or without negative control (Ctl), 10 μM of chloroquine (CQ) or 5mM of 3-MA for 4 h before cell harvest for immunoblotting against LC3 and FLAG. (**B**) A549 cells were infected with IFV A/PR8/8/34 (PR8) virus or NS1-deficient PR8 virus (PR8dNS1) at an MOI of 1 for 24 h. Cells were harvested and whole cell lysates (WCL) and mitochondrial fraction (Mito) were collected. Immunoblot was performed for LC3. TOM20 served as mitochondrial marker, whereas Calnexin was used as a negative marker of mitochondria. Densitometry quantification of LC3 is shown in numbers. RE: relative expression. (**C**) A549 cells were transfected with empty vector (EV) or NS1-FLAG encoding plasmids, along with vectors expressing mito-dsRED (red) and Parkin-GFP (green). Cells were treated with 10 μM of CCCP for 2 h. NS1-FLAG was detected using mouse anti-FLAG tag antibodies, followed by a goat anti-mouse secondary antibody, Cascade Blue. Scale bar = 20 μm. % cells with Parkin-GFP puncta in their mitochondria were calculated and are presented in the graph on the right. At least 100 cells were counted. ** *p* < 0.01 vs. EV-transfected CCCP-treated cells. (**D**) A549 cells were infected with PR8 or PR8dNS1 at an MOI of 1 and analyzed at 24 hpi. The cells were subsequently stained with MitoTracker Green (500 nM) and LysoTracker Red (75 nM). Scale bar = 20 μm. % cells with LysoTracker and MitoTracker colocalization were calculated and are presented in the graph. *** *p* < 0.001 vs. mock-treated cells, # *p* < 0.05 vs. PR8-infected cells. (**E**) Confocal analysis of HeLa cells expressing mt-Keima is shown. Cells were transfected with either empty vector (EV) or NS1-FLAG plasmid and treated with 25 μM CCCP or 10 μM CQ for 2 h. The emission signal obtained after excitation with the 488-nm laser is shown in green, and that obtained after excitation with the 561-nm laser is shown in red. Scale bar = 10 μm. The Zeiss ZEN software was used for quantitative colocalization analysis to demonstrate the changes in pH-dependent fluorescence. ** *p* < 0.01 vs. EV-transfected CCCP-treated cells.

In addition to downregulating apoptosis, NS1 is known to upregulate autophagy [15]. To confirm the role of NS1 enhancing autophagy, we first examined if autophagy flux is affected by NS1. Chloroquine (CQ) is a well-known inhibitor of autophagy flux [32], as evidenced by enhanced level of LC3-II protein, whereas 3-MA inhibits autophagy initiation. Figure 1A shows that NS1 expression leads to enhanced disruption of autophagy flux triggered by CQ. To investigate a possible role of NS1 affecting mitochondrial dynamics and mitophagy, we then examined whether NS1 is possibly associated with mitochondria. A549 cells were infected with either IFV A/Puerto-Rico/8/34 (H1N1) (PR8) virus or PR8 virus with NS1 deletion (PR8dNS1) virus and immunoblots of whole cell lysates vs. mitochondrial fraction were performed. Although NS1 protein was not detected in mitochondria, delNS1 infection led to decreased LC3 II/I levels in mitochondrial fraction compared with PR8 virus infection, suggesting a potential role of NS1 modulating mitophagy (Figure 1B).

Next, the transfection efficiency of NS1 plasmid in A549 cells was measured by immunofluorescence microscopy (Supplementary Figure S2). CCCP is a protonophore that disrupts mitochondrial membrane potential and induces mitophagy [21]. The effect of NS1 overexpression on CCCP-induced mitophagy was analyzed by confocal microscopy following IFV NS1 transfection. A549 cells were transfected with empty vector (EV) or NS1-FLAG plasmids along with mito-dsRED and Parkin-GFP expression vectors (Figure 1C). We observed that NS1-expressing cells showed a significantly increased number of cells with mitochondrial Parkin punctate forms. These data suggest that IFV NS1 can enhance parkin-dependent mitophagy. Next, A549 cells were infected with either PR8 or PR8dNS1 virus and were stained with LysoTracker Red and MitoTracker Green, which are both fluorescent probes that are widely used for staining lysosomes and mitochondria, respectively, in viable cells. Co-localization of signals from LysoTracker and MitoTracker following IFV infection in A549 cells was determined and presented as % cells containing mitolysosomes. Compared with 48.8% cells containing mitolysosomes in PR8-infected cells, PR8dNS1 virus infection resulted in less than 12.5% of cells containing mitolysosomes, indicating the essential role of NS1 in mitophagy induction (Figure 1D).

Previous reports suggest that mitophagy can be quantified using mt-Keima, a pH-sensitive, dual-excitation ratiometric fluorescent protein that also exhibits resistance to lysosomal proteases [19,20,21,33]. At the physiological pH of mitochondria (pH 8.0), shorter-wavelength excitation predominates and activation of mitophagy can lead to the formation of acidic lysosomes (pH 4.5); mt-Keima then undergoes a gradual shift to longer-wavelength excitation. For quantitative analysis of mitophagy using mt-Keima, the ratio of the area of lysosomal (red) signals to mitochondrial (green) signals can be used as a measurement of the lysosomal delivery of mitochondria. As shown in Figure 1E, IFV NS1 expression resulted in significant enhancement of mitophagy levels following CCCP treatment, compared with those in EV-transfected cells.

### 3.2. Mitophagy Induction via CCCP Treatment Increases IFV Replication

**Figure 2:**
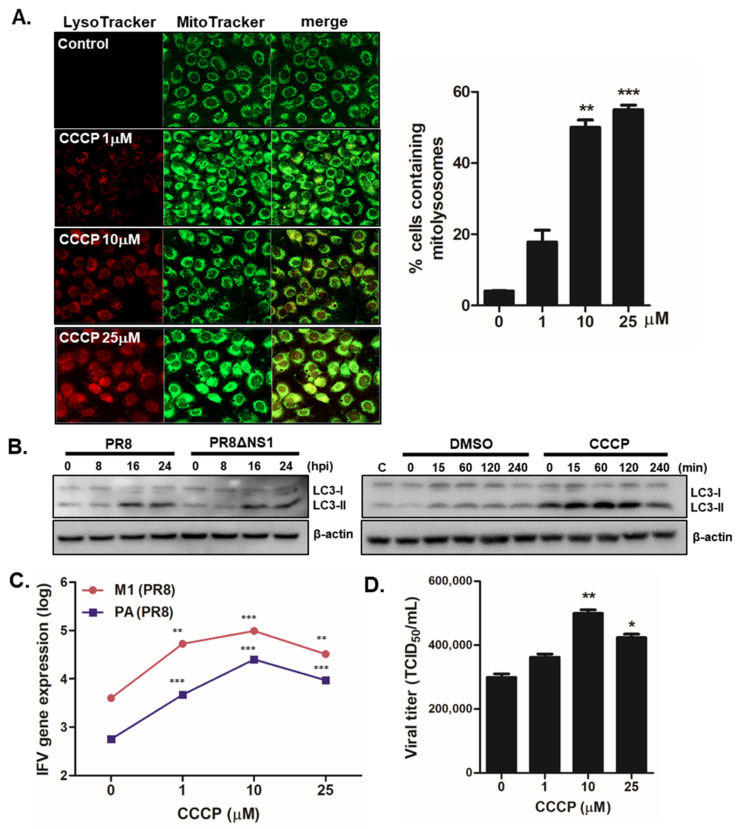
CCCP-mediated mitophagy activation enhances viral replication in A549 cells (**A**) Cells were treated with various concentration of CCCP for 2 h and subsequently stained with MitoTracker Green (500 nM) and LysoTracker Red (75 nM). Scale bar = 20 μm. % cells containing mitolysosomes were measured and are presented in the graph. ** *p* < 0.01; *** *p* < 0.001, vs. DMSO-treated cells. (**B**) A549 cells were infected with PR8 or PR8dNS1 at an MOI of 1 for indicated times (left) or were treated with 25 μM CCCP for indicated times (right). Immunoblot analysis of LC3 is shown. (**C**) Cells were pretreated with different concentrations of CCCP for 2 h and infected with PR8 virus at MOI of 1 for 24 h. IFV matrix protein 1 (M1) and polymerase protein (PA) copy numbers were quantitated by qRT-PCR, normalized, and analyzed (means ± SD; n = 3). * *p* < 0.05; ** *p* < 0.01; *** *p* < 0.001, vs. DMSO-treated cells. (**D**) Viral titers were measured using the TCID50 assay and expressed as TCID50/mL (means ± SD; n = 3).

The effect of mitophagy induction via CCCP treatment on IFV replication was measured by the following experiment. First, no cytotoxicity of 25 μM or less CCCP has been confirmed by cell viability assay (Supplementary Figure S3A). As expected, CCCP treatment in A549 cells led to mitophagy activation, as indicated by confocal microscopy and immunoblot analysis for detecting LC3 expression (Supplementary Figure S3B, Figure 2A,B). Increased cells containing mitolysosomes were detected by co-localization of signals from LysoTracker and MitoTracker following CCCP treatment, while there was a time-dependent increase in LC3-II expression following CCCP treatment and IFV infection. To further characterize whether CCCP could affect viral titers, A549 cells were pre-incubated with different doses of CCCP and infected with PR8 virus for 8 h. IFV matrix protein 1 (M1) and polymerase protein (PA) gene expression levels were then measured by qRT-PCR. We demonstrated that CCCP treatment significantly upregulated PR8 virus replication in A549 cells in a dose-dependent manner (Figure 2C). Next, we performed a TCID50 assay and found augmented viral titers following CCCP treatment (Figure 2D).

### 3.3. IFV NS1 Induces Mitochondrial Fragmentation

**Figure 3:**
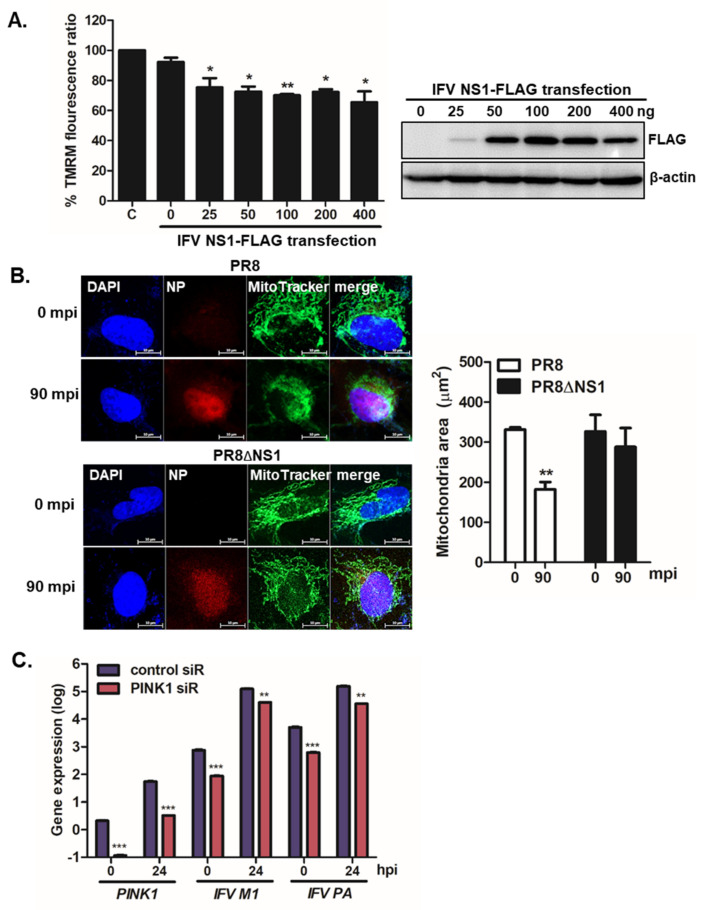
IFV NS1 depolarizes MMP and enhances mitochondrial fragmentation. (**A**) A549 cells were transiently transfected with the indicated amount of NS1-FLAG plasmid for 24 h and were then treated with CCCP for 2 h. Relative tetramethylrhodamine methyl ester (TMRM) intensities in each group of live cells were measured using the Varioskan LUX multiplate reader (on the left). C indicates treatment with CCCP alone. TMRM fluorescence from CCCP (**C**) alone-treated cells was set to 100% and other samples (NS1-transfected cells treated with CCCP) were represented by % TMRM fluorescence ratio. Efficient transfection of IFV NS1 is shown by immunoblot analysis (on the right). (**B**) A549 cells were infected with PR8, or PR8dNS1 virus for 90 min (MOI = 1). Anti-IFV nucleoprotein (NP) antibodies were used to detect NP protein (red) and MitoTracker Green (green) was used to observe mitochondrial areas; minutes post IFV infection (mpi); Scale bar = 10 μm. Quantification of mitochondrial area is shown in the graph (n = 100). ** *p* < 0.01; vs. PR8-infected cells at 0 mpi (**C**) A549 cells transfected with either control or PINK1 siRNA were infected with PR8 at an MOI of 1 for the indicated time periods. The qRT-PCR results indicate the expression levels of PINK1, IFV M1, and IFV PA. * *p* < 0.05; ** *p* < 0.01; *** *p* < 0.001 vs. control siRNA-transfected cells.

Mitophagy is preceded by the loss of mitochondrial membrane potential (MMP), and mitochondrial fission is required to remove damaged mitochondria [3]. To characterize the potential role of IFV NS1 during mitophagy, we examined the ability of the viral protein to affect CCCP-mediated MMP loss. As demonstrated in Figure 3A, IFV NS1 expressing cells led to a significant loss of MMP triggered by CCCP treatment, as shown by reduced signals of tetramethylrhodamine methyl ester (TMRM), a small cationic fluorescent indicator that accumulates in mitochondria purely based on the ΔΨm. Next, to further characterize whether IFV NS1 is involved in mitochondrial fragmentation during infection, we compared the mitochondrial morphology in PR8 vs. PR8dNS1 virus-infected cells. Figure 3B shows that active mitochondrial fission was observed inPR8 virus-infected cells, whereas elongated mitochondrial networks were maintained in PR8dNS1 virus-infected cells even at 90 mpi.

PTEN-induced kinase 1 (PINK1), along with Parkin, is a major player in mitophagy and an upstream regulator of Parkin function. Therefore, to evaluate the effect of PINK1 knockdown on IFV replication, we transfected A549 cells with siRNAs specific to the control or PINK1. After 24 h, *PINK1*, *IFV M1*, and *IFV PA* mRNA levels were compared with those in control siRNA-transfected cells. Quantitative RT-PCR data revealed efficient knockdown of PINK1, and a significant attenuation of *IFV M1* and *PA* gene expression following PINK1 knockdown, highlighting the importance of PINK1 expression and mitophagy induction for productive viral replication (Figure 3C).

### 3.4. ULK1 Translocates to Mitochondria upon IFV Infection and Supports Viral Replication

**Figure 4:**
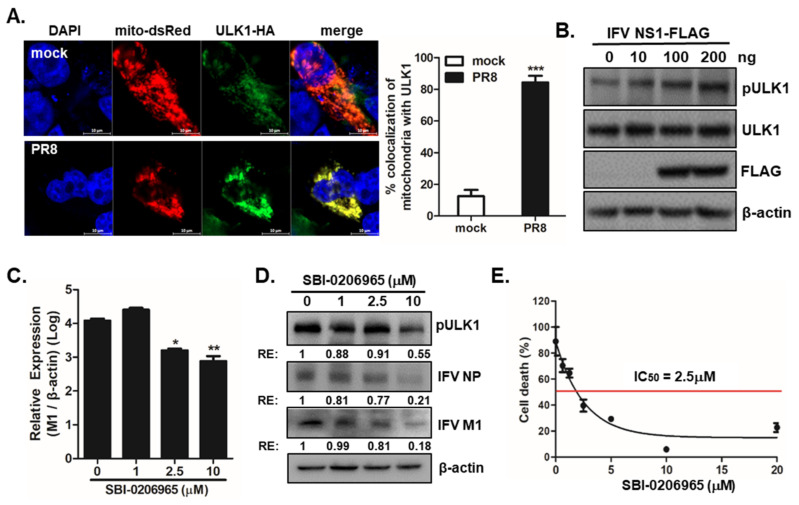
ULK1 is important for mitophagy and IFV replication. (**A**) A549 cells transfected with mito-dsRED (red) and ULK1-HA (green) plasmid were infected with PR8 virus at an MOI of 1 for 24 h. ULK1 was detected with rabbit anti-HA tag antibody, followed anti-rabbit FITC-conjugated antibody. Scale bar = 10 μm. (**B**) Phosphorylation of ULK1 was evaluated in A549 cells transfected with IFV NS1-FLAG plasmid for 24 h and treated with CCCP for 2 h by immunoblot analysis. (**C**–**E**) A549 cells were pretreated with different concentrations of SBI and infected with PR8 virus at an MOI of 1 for 24 h. (**C**) The IFV M1 copy number was quantitated by qRT-PCR, normalized, and analyzed (means ± SD; n = 3). * *p* < 0.05; ** *p* < 0.01; ** *p* < 0.001, vs. DMSO-treated cells. (**D**) IFV NP and IFV M1 protein expression was measured by immunoblot analysis following treatment with the indicated doses of SBI (**E**). The 50% inhibitory concentration (IC50) of SBI was determined using the 4-parameter logistic nonlinear regression model equation in GraphPad Prism software.

Unc-51-like autophagy activating kinase 1 (ULK1) is a serine/threonine kinase required for early autophagosome formation; it is associated with damaged mitochondria and is recruited to the mitochondria by binding to FUNDC1 [34]. To evaluate the effect of ULK1 on IFV-mediated mitophagy, we measured whether IFV infection promotes translocation of ULK1 to mitochondria by confocal microscopy analysis. Figure 4A shows that ULK1 translocates to the mitochondria in IFV-infected cells. Moreover, NS1 overexpression led to increased phosphorylation of ULK1 by immunoblot analysis (Figure 4B). Next, we used an ULK1 specific inhibitor (SBI-0206965, SBI) in the subsequent experiments. IFV M1 expression was measured following SBI treatment in PR8-infected A549 cells and found that SBI treatment, at a starting concentration of 2.5 μM, significantly reduced the expression of IFV M1 transcripts (Figure 4C). In agreement with these data, immunoblot analysis showed that IFV NP and M1 protein expression was also suppressed upon SBI treatment in a dose-dependent manner (Figure 4D). We also assessed the inhibitory concentration 50 (IC50) of PR8 virus following SBI treatment in A549 cells; the IC50 of SBI was found to be 2.5 μM (Figure 4E).

### 3.5. IFV NS1 Induces the Expression of the Mitophagy Receptor BNIP3, Which Is Essential for Viral Replication

**Figure 5:**
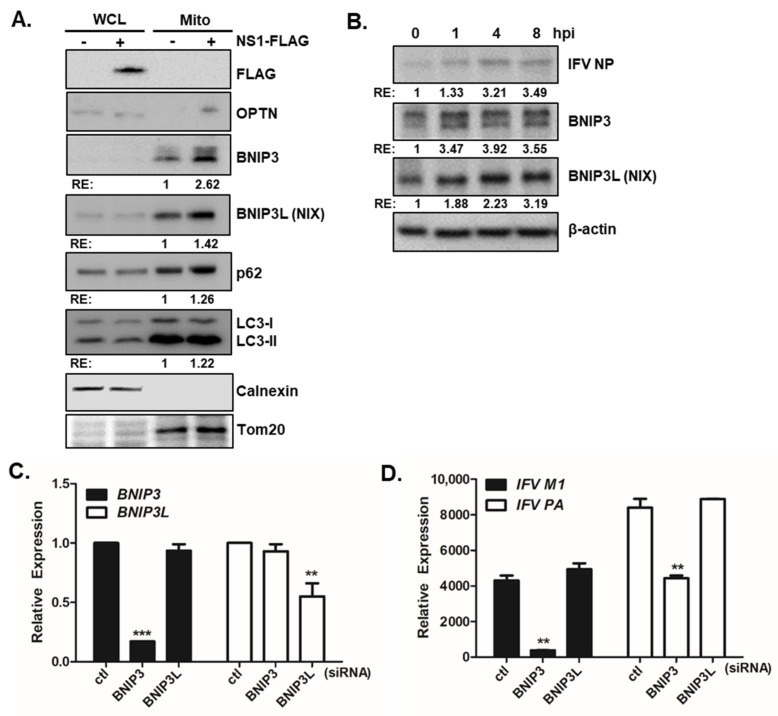
IFV NS1 upregulates mitochondrial BNIP3 expression, which is essential for viral replication. (**A**) Immunoblot analysis shows whole-cell lysates (WCL) and mitochondrial fractions (Mito) derived from A549 cells transfected with either empty vector (−) or NS1-FLAG (+) plasmids. The relative band intensities were calculated using ImageJ software and are shown as numbers in the column. (**B**) A549 cells were infected with PR8 virus at an MOI of 1 for the indicated times. Protein expression levels of BNIP3 and BNIP3L were determined by immunoblot analysis. A representative of three independent experiments are shown. (**C**,**D**) A549 cells transfected with either control or BNIP3 or BNIP3L siRNA were infected with PR8 virus at a multiplicity of infection (MOI) of 1 for the indicated time periods. qRT-PCR results indicated the expression levels of *BNIP3*, *BNIP3L*, *IFV M1* and *IFV PA*. ** *p* < 0.01; *** *p* < 0.001 vs. the control siRNA-transfected cells.

To further determine whether NS1 upregulates specific mitophagy receptors, we performed mitochondrial fractionation from the empty vector or NS1-transfected cells following CCCP treatment. IFV NS1 expression can lead to increased expression levels of mitophagy receptors such as optineurin (OPTN), BCL2-interacting protein 3 (BNIP3), and BNIP3-like homolog (BNIP3L/NIX) in the mitochondrial fractions of A549 cells (Figure 5A). Besides its role in xenophagy, OPTN has been identified as a primary autophagy receptor that translocates to the mitochondria during mitophagy and has been implicated in cancer and neurological disorders [35]. BNIP3 and BNIP3L/NIX have dual function in both cell death and autophagy, in particular, both are known to recruit autophagosomes to mitochondria via its direct interaction with LC3 and induce mitophagy in Parkin-deficient cells [36,37,38].

A recent study by O’Sullivan et al., showed that BNIP3 and BNIP3L are essential for natural killer cell survival after viral infection [39]. We also detected increased expression of BNIP3 and BNIP3L following IFV infection in A549 cells (Figure 5B). Given that IFV NS1 upregulates mitophagy receptors such as BNIP3 and BNIP3L, we further examined the potential role of BNIP3 and BNIP3L during IFV infection. Quantitative RT-PCR was used to determine the viral copy number following *BNIP3* and *BNIP3L* siRNA transfection. Figure 5C confirms that the transfection of siRNAs specific to BNIP3 or BNIP3L led to efficient knockdown. Quantitative RT-PCR data indicated that the deficiency in *BNIP3* expression led to a significant reduction in *IFV M1* and *PA* copy numbers, whereas lack of BNIP3L expression did not affect IFV gene expression (Figure 5D). We also showed that BNIP3 knockdown in A549 cells led to a significant reduction in the percentage of cells with GFP-LC3 puncta in mitochondria, indicating the importance of BNIP3 in enhancing CCCP-mediated mitophagy (Supplementary Figure S4).

## 4. Discussion

Mitochondria are involved in a variety of cellular metabolic processes, and there are several quality control mechanisms that can maintain mitochondrial homeostasis. Mitophagy serves as one of these mechanisms to selectively remove damaged mitochondria. Increasing evidence shows an important role for mitophagy in the promotion of viral infections, and the mitophagic process can be regulated by multiple viruses [4,5,6,21,40,41,42,43,44,45,46]. For example, the matrix protein (M) of human parainfluenza virus type 3 translocates to the mitochondria and interacts with Tu translation elongation factor mitochondrial (TUFM). M-mediated mitophagy does not require the Parkin-PINK1 pathway; instead, the interaction between M and the LC3 protein mediates autophagosome formation.

Multiple studies have shown that autophagy promotes IFV polymerase activity, thereby increasing viral RNA synthesis as well as upregulation of viral progeny production [16,17,18]. Although IFVs have also been shown to induce mitophagy [47], detailed mechanisms of how IFV affects mitophagy remain unclear. In this study, we observed that CCCP-mediated mitophagy facilitates IFV replication. In contrast, inhibition of Parkin-mediated mitophagy by silencing PINK1 results in the downregulation of IFV replication (Figure 3). In accordance with our finding, Perot et al., revealed that IFV-mediated autophagy downregulates IFN induction and signaling [48]. Furthermore, a recent study by Wang et al., suggested that PB1-F2 of IFV interacts and colocalizes with TUFM to induce mitophagy, which contributes to the degradation of mitochondrial antiviral signaling protein (MAVS) in order to suppress IFN production [6]. Our study corroborates these findings, stating that IFV NS1 alters mitochondrial dynamics, leading to the depolarization of mitochondrial membrane potential, induction of mitochondrial fragmentation, and enhancement of Parkin-mediated mitophagy. Infection with PR8dNS1 virus resulted in impaired mitochondrial fragmentation, subsequent mitolysosomal formation, and mitophagy induction, suggesting an important role for IFV NS1 in mitophagy. Given that mitophagy has recently been described to inhibit the antiviral IFN pathway [49], it is highly possible that the role of NS1 in mitophagy may directly or indirectly affect the function of NS1 in evading host innate immunity.

The role of IFV NS1 during autophagy has been reported by several groups. First, Zhirnov et al., demonstrated that NS1 upregulates autophagy indirectly by stimulating the synthesis of M2 and HA, which in turn initiates autophagosome formation [15]. Accordingly, PR8dNS1 virus infection led to diffused LC3 accumulation, resulting in decreased autophagosome formation. In contrast, Kuroki et al., recently reported that IFV NS1 blocks JNK1-dependent autophagosome formation regulated by Rab11a-mediated endosome recycling [50]. Notably, our studies revealed that NS1 can increase mitochondrial fragmentation and mitophagy although NS1 was not detected in mitochondrial fraction, thus the exact mechanisms by which NS1 would affect mitochondrial dynamics and mitophagy need to be fully determined. Given that NS1 interacts specifically with the nucleoprotein of ribonucleoprotein complexes [51], it is possible that NS1 may affect another IFV non-structural proteins, such as PB1-F2, to affect mitophagy. In fact, the PB1-F2 protein translocates to the mitochondria, accelerates mitochondrial fragmentation, and impairs innate immunity via interaction with TUFM [6]. Meanwhile, the NP and M2 proteins also induce autophagy and interact with LC3 [18], therefore, it will be interesting to further study whether NS1 affects mitophagy via interaction with NP or M2 proteins.

Here, our study unveils a new finding that IFV infection promotes the translocation of ULK1 to mitochondria, and NS1 overexpression leads to phosphorylation of ULK, which is important for modulating mitophagy and virus replication (Figure 4). ULK1 is a mammalian homolog of *ATG1* gene, which is an autophagy initiating kinase, and has been identified to play an important role in mitophagy [34,52]. ULK1 can be phosphorylated by both adenosine-monophosphate-activated protein kinase (AMPK) and mTORC1 [53,54]. Although there is mounting evidence to suggest a significant role of cellular kinases, such as PI3K or PKC, for regulating IFV infection [55], a potential role of ULK1 contributing to mitochondrial dynamics during IFV infection needs further investigation. We and other groups previously showed that IFV infection in vitro and in vivo can lead to activation of AMPK [56,57]. Further studies on the role of AMPK-ULK1 signaling in control of mitophagy will facilitate improved understanding of IFV replication in host cells.

Another important observation in the current study is that IFV NS1 upregulates BNIP3 expression in mitochondria and BNIP3 is important for IFV replication. A previous report suggested that IFV-mediated mitophagy may be independent of Parkin-dependent pathways, and that it was interesting to further identify the role of other mitophagy receptors during IFV infection [47]. BNIP3 and BNIP3L/NIX belong to Bcl-2 family, which induce both cell death and autophagy [36]. Additional functions of BNIP3 and BNIP3L/NIX have recently been identified. For example, human herpesvirus 8-encoded viral interferon regulatory factor 1 was found to promote mitochondrial clearance by activating BNIP3L/NIX-mediated mitophagy, supporting viral replication [58]. Moreover, a recent study by O’Sullivan et al. [39] demonstrated that BNIP3 and BNIP3L/NIX mediated the removal of reactive oxygen species and depolarized mitochondria via mitophagy to induce natural killer cell memory formation after viral infection. Despite the emerging evidence suggesting BNIP3′s involvement in mitophagy, the regulatory mechanisms of Bnip3 in mitochondrial dysfunction and mitophagy are still poorly understood. Zhang et al., reported that BNIP3 interacts with PINK1 to promote the translocation of Parkin to mitochondria [59]. Our findings raise an important possibility that NS1 may promote BNIP3 expression, which may interact with LC3 to target dysfunctional mitochondria to autophagosomes, suggesting a possible role of BNIP3 facilitating the activation of mitophagy.

Taken together, our data provide the first evidence that IFV NS1 is important for triggering cascades involving the regulation of mitochondrial dynamics and mitophagy possibly via ULK1 and BNIP3. Future experiments expanding our knowledge on the role of BNIP3 and other mitophagic receptors in the pathogenesis of IFV and other viruses will enhance our knowledge regarding the host factors determining IFV susceptibility. Collectively, these findings provide a foundation for further studies to investigate the potential of mitochondrial fission inhibitors or mitophagy inhibitors to serve as possible candidate therapeutic agents against viral infection.

## Figures and Tables

**Table 1:** Primer sequences used in this study.

Gene	Primer-Forward	Primer-Reverse
IFV M1	ATGAGYCTTYTAACCGAGGTCGAAACG	TGGACAAANCGTCTACGCTGCAG
IFV PA	CGGTCCAAATTCCTGCTGAT	CATTGGGTTCCTTCCATCCA
PINK1	GGACACGAGACGCTTGCA	TTACCAATGGACTGCCCTATCA
BNIP3	CAGGGCTCCTGGGTAGAACT	CTACTCCGTCCAGACTCATGC
BNIP3L	TTGGATGCACAACATGAATCAGG	TCTTCTGACTGAGAGCTATGGTC
β-actin	GAGCACAGAGCCTCGCCTTT	ACATGCCGGAGCCGTTGTC

**Table 2:** siRNA sequences used in this study.

siRNA	Sense	Antisense
PINK1	CAGACAUCUGAAAAGUGAA	UUCACUUUUCAGAUGUCUG
BNIP3	GAGAGAAAAACAGCUCACA	UGUGAGCUGUUUUUCUCUC
BNIP3L	CAGUUGACAGCGUUCUGAA	UUCAGAACGCUGUCAACUG

## Data Availability

Not applicable.

## Supplementary Materials

The following are available online at https://www.mdpi.com/article/10.3390/v13091845/s1, Figure S1: IFV NS1 inhibits apoptosis, Figure S2: Transfection efficiency of NS1-FLAG plasmid is shown by immunofluorescence staining, Figure S3: Effect of CCCP on mitophagy induction is confirmed. Figure S4: BNIP3 enhances CCCP-mediated mitophagy.
