# Supplementary material for: Nonstructural Protein NS1 of Influenza Virus Disrupts Mitochondrial Dynamics and Enhances Mitophagy via ULK1 and BNIP3
Source: Viruses. 2021 Sep 15;13(9):1845. doi: 10.3390/v13091845 (PMC8473137; doi:10.3390/v13091845)
Supplement: Supplementary file 1 [file viruses-13-01845-s001.zip › viruses-1360249-supplementary.pdf]

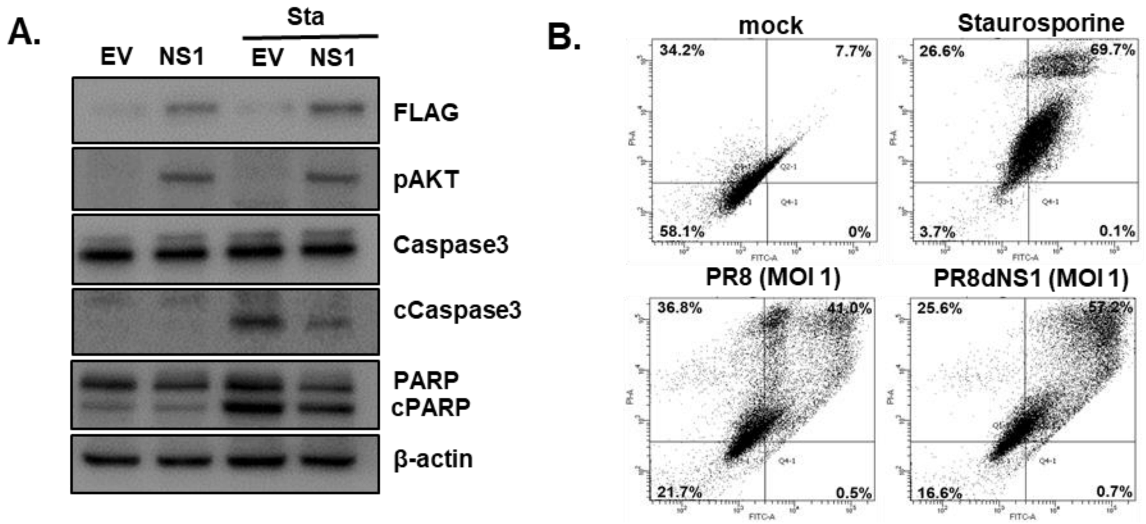


**Supplementary Figure S1. IFV NS1 inhibits apoptosis** (A) A549 cells were transfected with empty vector (EV) or IFV NS1-FLAG tagged plasmid for 24 h. Staurosporine (Sta) 1uM was added to the cells, followed by incubation for 4 h to induce apoptosis. The protein levels of phospho-AKT, cleaved caspase 3, and PARP were analyzed by immunoblot analysis. Actin was used as the loading control. The blot shown is representative of three independent experiments. (B) Flow cytometry analysis of Annexin V-FITC (x-axis) and propidium iodide (PI) (Y-axis) staining in A549 cells infected with either IFV A/PR8/8/34 (PR8) or delNS1 virus (PR8dNS1) at an MOI of 1 for 8 h. Staurosporine 1uM was added to mock-infected cells for 4 hours as a positive control.

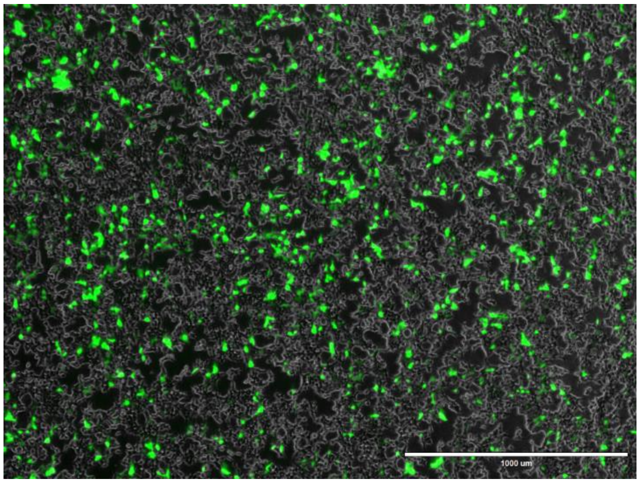


**Supplementary Figure S2. Transfection efficiency of NS1-FLAG plasmid is shown by immunofluorescence staining**

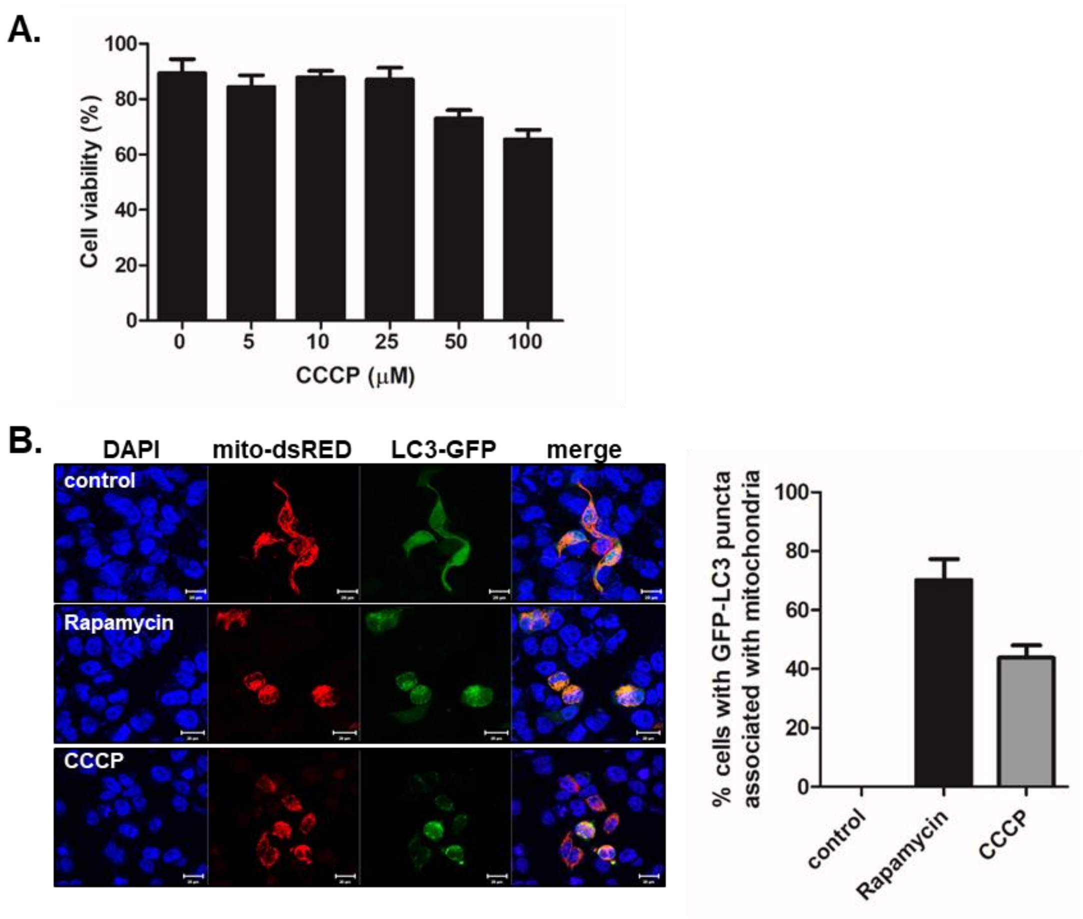


**Supplementary Figure S3. Effect of CCCP on mitophagy induction is confirmed.** (A) A549 cells were treated with various concentration of CCCP for 2 hours. Cell survival was monitored by CellTiter-Glo® Luminescent Cell Viability Assay (Promega, Madison, WI) as per manufacturer's instructions. (B) A549 cells were transfected with vectors expressing mito-dsRED (red) and LC3-GFP (green). Cells were treated with 100nM rapamycin for 8 h or 25 μM of CCCP for 2 h. Scale bar = 20μm. % cells with LC3-GFP puncta in their mitochondria were calculated and are presented in the graph on the right. At least 100 cells were counted.

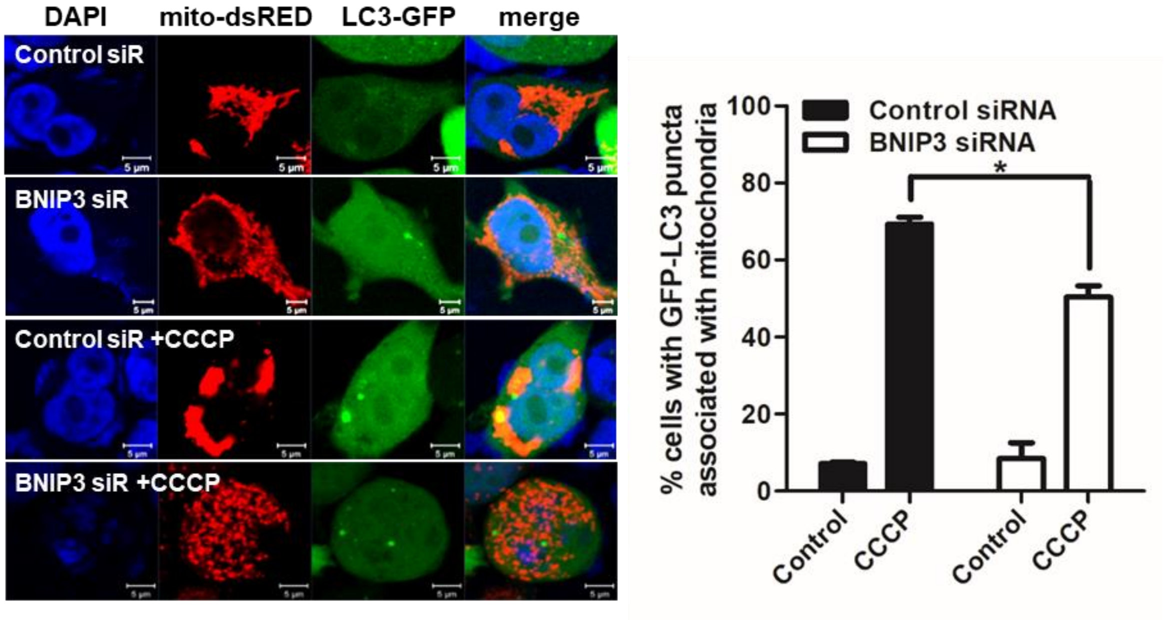


**Supplementary Figure S4. BNIP3 enhances CCCP-mediated mitophagy.** A549 cells were transfected with mito-dsRED (red) and LC3-GFP (green), followed by co-transfection with control or BNIP3 siRNAs for 24 h. On the next day, cells were treated with CCCP for 2 h. Scale bar =5 µm. % cells with GFP-LC3 puncta in mitochondria were quantified and represented in the graph. $*p < 0.05$; $**p < 0.01$; $***p < 0.001$ versus control siRNA-transfected cells.
